# Supplementary material for: MetaRibo-Seq measures translation in microbiomes
Source: Nat Commun. 2020 Jun 29;11:3268. doi: 10.1038/s41467-020-17081-z (PMC7324362; doi:10.1038/s41467-020-17081-z)
Supplement: Supplementary file 10 — Supplementary Data 7 [file 41467_2020_17081_MOESM10_ESM.zip › File2/Confidence_High_Taxonomy/275966_out.krona.html]

Javascript must be enabled to view this page.

members
magnitude
magnitudeUnassigned
count
unassigned
taxon
rank

275966\_out

173

173
2
superkingdom

phylum
173
976

class
200643
173

order
171549
173

1
2005525
family

1
375288
genus


SRS046717\_contig\_number\_5218
species
46503
1

815
169
family

1
169

SRS054352\_contig\_number\_16004
genus
816

821
1
species

SRS1055076\_contig\_number\_16882


SRS015782\_contig\_number\_45803SRS016954\_contig\_number\_18165SRS016989\_contig\_number\_14830SRS065176\_contig\_number\_561
4
674529
species

species
6
1739319

SRS016095\_contig\_number\_29490SRS019445\_contig\_number\_15636SRS076929\_contig\_number\_27154SRS076929\_contig\_number\_contig-100\_1255.149081SRS104975\_contig\_number\_19944SRS893170\_contig\_number\_10627


SRS012969\_contig\_number\_25595SRS015190\_contig\_number\_16759SRS022137\_contig\_number\_19561SRS023526\_contig\_number\_38510SRS043411\_contig\_number\_17657SRS055982\_contig\_number\_16623
2292951
6
species

species
67
2302925

SRS011239\_contig\_number\_contig-100\_159.117270SRS012849\_contig\_number\_contig-100\_525.200722SRS013382\_contig\_number\_contig-100\_3435.3436SRS014923\_contig\_number\_29628SRS016018\_contig\_number\_2585SRS016056\_contig\_number\_contig-100\_175.49528SRS016095\_contig\_number\_contig-100\_1314.167981SRS017103\_contig\_number\_contig-100\_607.126485SRS017247\_contig\_number\_10530SRS017247\_contig\_number\_contig-100\_1221.67366SRS017307\_contig\_number\_contig-100\_3561.136313SRS018541\_contig\_number\_contig-100\_642.92375SRS018817\_contig\_number\_21560SRS018888\_contig\_number\_4959SRS019693\_contig\_number\_7750SRS020328\_contig\_number\_contig-100\_2135.204327SRS020508\_contig\_number\_contig-100\_363.18101SRS024663\_contig\_number\_13321SRS042628\_contig\_number\_contig-100\_640.200296SRS043841\_contig\_number\_10949SRS044535\_contig\_number\_25438SRS046068\_contig\_number\_833SRS049446\_contig\_number\_8144SRS049959\_contig\_number\_contig-100\_422.268908SRS050422\_contig\_number\_29020SRS050925\_contig\_number\_27147SRS051882\_contig\_number\_19867SRS052576\_contig\_number\_contig-100\_164.165SRS054905\_contig\_number\_21081SRS056259\_contig\_number\_2695SRS056537\_contig\_number\_47747SRS062427\_contig\_number\_3700SRS063518\_contig\_number\_39321SRS065397\_contig\_number\_9757SRS074964\_contig\_number\_5506SRS075773\_contig\_number\_37113SRS075878\_contig\_number\_23424SRS075984\_contig\_number\_27229SRS076804\_contig\_number\_20316SRS077552\_contig\_number\_23497SRS077849\_contig\_number\_23890SRS078242\_contig\_number\_contig-100\_1700.179648SRS098073\_contig\_number\_16963SRS098571\_contig\_number\_52035SRS098881\_contig\_number\_17503SRS101376\_contig\_number\_17462SRS103987\_contig\_number\_26579SRS1041129\_contig\_number\_14183SRS1041141\_contig\_number\_13581SRS1041146\_contig\_number\_contig-100\_74.18553SRS1041164\_contig\_number\_6628SRS104311\_contig\_number\_45916SRS104327\_contig\_number\_3088SRS1055083\_contig\_number\_contig-100\_1422.82960SRS142542\_contig\_number\_20985SRS142599\_contig\_number\_24707SRS142980\_contig\_number\_contig-100\_1907.53910SRS143181\_contig\_number\_12178SRS144537\_contig\_number\_19040SRS144603\_contig\_number\_14698SRS145308\_contig\_number\_5057SRS147022\_contig\_number\_868SRS148424\_contig\_number\_34440SRS148817\_contig\_number\_14067SRS149879\_contig\_number\_24620SRS893358\_contig\_number\_13150SRS971276\_contig\_number\_22474


SRS015579\_contig\_number\_26638SRS018313\_contig\_number\_11715SRS018936\_contig\_number\_11480SRS024625\_contig\_number\_16031SRS047044\_contig\_number\_12234SRS052027\_contig\_number\_20047SRS053398\_contig\_number\_22631SRS053573\_contig\_number\_905SRS053649\_contig\_number\_20194SRS065504\_contig\_number\_contig-100\_438.210913SRS077231\_contig\_number\_contig-100\_1225.151260SRS145308\_contig\_number\_contig-100\_1272.34529SRS148721\_contig\_number\_49519SRS893369\_contig\_number\_5409SRS893378\_contig\_number\_23480
15
28116
species


SRS019601\_contig\_number\_contig-100\_182.169203SRS046810\_contig\_number\_contig-100\_22.23SRS049896\_contig\_number\_contig-100\_824.199402SRS050801\_contig\_number\_2689SRS1041116\_contig\_number\_1340SRS1041138\_contig\_number\_6097SRS142980\_contig\_number\_contig-100\_961.52964SRS893259\_contig\_number\_5237SRS893288\_contig\_number\_3785SRS893342\_contig\_number\_3739
species
457390
10

1
371601
species

SRS013687\_contig\_number\_contig-100\_313.184148


SRS018427\_contig\_number\_23618SRS021219\_contig\_number\_13033SRS148253\_contig\_number\_3528
817
3
species


SRS013638\_contig\_number\_11475SRS013800\_contig\_number\_27499SRS014287\_contig\_number\_16023SRS014855\_contig\_number\_11546SRS015854\_contig\_number\_21194SRS016897\_contig\_number\_1731SRS017859\_contig\_number\_2608SRS019397\_contig\_number\_9746SRS020622\_contig\_number\_3318SRS021153\_contig\_number\_3732SRS023346\_contig\_number\_4263SRS024331\_contig\_number\_38500SRS024435\_contig\_number\_36104SRS045004\_contig\_number\_22780SRS045645\_contig\_number\_2978SRS046712\_contig\_number\_1558SRS048870\_contig\_number\_17590SRS051610\_contig\_number\_7950SRS052697\_contig\_number\_37938SRS057717\_contig\_number\_889SRS063928\_contig\_number\_900SRS075821\_contig\_number\_23920SRS076756\_contig\_number\_19421SRS077454\_contig\_number\_1579SRS077589\_contig\_number\_7155SRS104084\_contig\_number\_10760SRS1041090\_contig\_number\_6004SRS140645\_contig\_number\_1552SRS142923\_contig\_number\_23131SRS143085\_contig\_number\_3929SRS143722\_contig\_number\_672SRS146764\_contig\_number\_17347SRS146812\_contig\_number\_49410SRS147272\_contig\_number\_1451SRS147377\_contig\_number\_2329SRS147977\_contig\_number\_3330SRS148091\_contig\_number\_12535SRS148970\_contig\_number\_1739SRS149325\_contig\_number\_16219SRS892882\_contig\_number\_16003SRS893373\_contig\_number\_24827
species
818
41


SRS016954\_contig\_number\_19289SRS018836\_contig\_number\_16385SRS019161\_contig\_number\_41880SRS020328\_contig\_number\_37347SRS024625\_contig\_number\_19266SRS042966\_contig\_number\_11714SRS046717\_contig\_number\_1552SRS049900\_contig\_number\_5218SRS050941\_contig\_number\_contig-100\_3.145459SRS058770\_contig\_number\_5055SRS064645\_contig\_number\_2248SRS076976\_contig\_number\_21051SRS100021\_contig\_number\_12064
46506
13
species


SRS142503\_contig\_number\_31216
28111
1
species

1
1853231
family

1
283168
genus

28118
1
species

SRS024075\_contig\_number\_10385

171550
2
family

genus
2
239759


SRS053398\_contig\_number\_26104
626932
1
species

28117
1
species

SRS145497\_contig\_number\_19954
